# Sir Henry Thompson's *Stricture of Urethra*

**Published:** 1885-06

**Authors:** 


*Stricture of the Urethra and Urinary Fistulæ.* Fourth Edition. Pp. 254. By Sir HENRY THOMPSON. London: J. & A. CHURCHILL. 1855.

How many men can say this of the fourth edition of their work?—"I have *reduced* the bulk of the work by upwards of a hundred pages." The pruning knife has not injured the vitality of the tree, nor impaired the quality of the fruit. In every respect, this edition is an improvement on the work which had already become a classic. Few surgical writers have surpassed Sir Henry Thompson in collecting and arranging facts; and, probably, none have excelled him in copious versatility in drawing therapeutical indications from these facts. But most of all do we admire his methods of classification and description. There is here none of the laxity of the mere bookmaker; each subject is clearly cut out from its neighbour, placed in its proper position, and described in simple, elegant, and concise language. All this means labour, bestowed, probably, with less stint than most of us would believe; and we are grateful to the writer for this labour, for it increases our power of understanding the subject, and lightens our efforts in studying it.

